# Analysis of RL electric circuits modeled by fractional Riccati IVP via Jacobi-Broyden Newton algorithm

**DOI:** 10.1371/journal.pone.0316348

**Published:** 2025-01-14

**Authors:** Mahmoud Abd El-Hady, Mohamed El-Gamel, Homan Emadifar, Atallah El-shenawy

**Affiliations:** 1 Department of Mathematics and Engineering Physics, Faculty of Engineering, Mansoura University, Mansoura, Egypt; 2 Department of Mathematics, Saveetha School of Engineering, Saveetha Institute of Medical and Technical Sciences, Saveetha University, Chennai, Tamil Nadu, India; 3 MEU Research Unit, Middle East University, Amman, Jordan; 4 Department of Mathematics, Hamedan Branch, Islamic Azad University, Hamedan, Iran; 5 Department of Mathematics, Faculty of Sciences, New Mansoura University, New Mansoura, Egypt; University of Porto Faculty of Engineering: Universidade do Porto Faculdade de Engenharia, PORTUGAL

## Abstract

This paper focuses on modeling Resistor-Inductor (RL) electric circuits using a fractional Riccati initial value problem (IVP) framework. Conventional models frequently neglect the complex dynamics and memory effects intrinsic to actual RL circuits. This study aims to develop a more precise representation using a fractional-order Riccati model. We present a Jacobi collocation method combined with the Jacobi-Newton algorithm to address the fractional Riccati initial value problem. This numerical method utilizes the characteristics of Jacobi polynomials to accurately approximate solutions to the nonlinear fractional differential equation. We obtain the requisite Jacobi operational matrices for the discretization of fractional derivatives, therefore converting the initial value problem into a system of algebraic equations. The convergence and precision of the proposed algorithm are meticulously evaluated by error and residual analysis. The theoretical findings demonstrate that the method attains high-order convergence rates, dependent on suitable criteria related to the fractional-order parameters and the solution’s smoothness. This study not only improves comprehension of RL circuit dynamics but also offers a solid numerical foundation for addressing intricate fractional differential equations.

## 1 Introduction

Recently, non-integer derivatives are used in ordinary differential equations to model various real-world events, for instance, fluid dynamics, visco-elastic damping, fluid flow tracing, electro-chemistry, electrical circuits, physics, the model of neurons in biology, voltage dividers, quantum mechanics, electromagnetism, hydrology, mathematical biology, etc [1–4]. Furthermore, fractional derivatives are widely renowned for their ability to efficiently govern models [5–10]. Recent developments in fractional calculus have broadened its applications in numerous scientific and engineering domains. A notable advancement is the introduction of novel fractional operators, like the Atangana-Baleanu fractional derivative [11], which enhance modeling efficacy for intricate systems exhibiting memory effects and non-local characteristics. Moreover, academics have devised advanced numerical approaches, such as spectral methods and fractional finite element methods, to effectively address fractional differential equations. These improvements have resulted in substantial breakthroughs in control systems, signal processing, image processing, and biotechnology. Fractional-order controllers have demonstrated the ability to boost the performance of dynamical systems, whilst fractional-order filters have enhanced noise reduction and signal denoising.

Mathematicians and physicists have worked hard to create strong numerical and analytical methodologies for obtaining the solutions of fractional differential equations. Numerical approaches have been frequently employed in the last two decades to get approximate solutions to applied and scientific problems such as finite differences, finite elements, boundary element techniques, etc. A summary of the essential techniques related to the approximate solution of various differential equations with fractional derivatives is displayed in Table 1.

**Table 1:** Summary of relevant papers concerning fractional differential equations.

Type of solution	Method	Author	Ref.
**Analytical**	Adomian decomposition approach (ADA)	Wang	[15]
	Variational iteration method (VIM)	Inc	[16]
	Homotopy analysis method (HAM)	Zhang et al.	[17]
	Modified homotopy perturbation method	Odibat et al.	[18]
	Laplace decomposition method (LDM)	Alchikh et al.	[19]
	Differential transform method (DTM)	Matinfar et al.	[20]
**Numerical**	Finite difference approximations	Meerschaert et al.	[21]
	Implicit difference approximation	Karatay et al.	[22]
	Finite element method	Li et al.	[23]
	Finite volume method	Zhang et al.	[24]
**Spectral techniques**	Jacobi spectral method	Sazmand et al.	[25]
		El-Gamel et al.	[26]
	Shifted fractional Jacobi collocation method	Abdelkawy et al.	[27]
	Cardinal B-spline functions	Lakestani et al.	[28]
	Haar wavelet operational matrix method	Lepik	[29]
	Shifted Chebyshev polynomials	Bhrawy et al.	[30]
	Multiscale Galerkin algorithm	Chen	[31]
	Chelyshkov-tau approach	El-Gamel et al.	[32]

**Fig 1:**
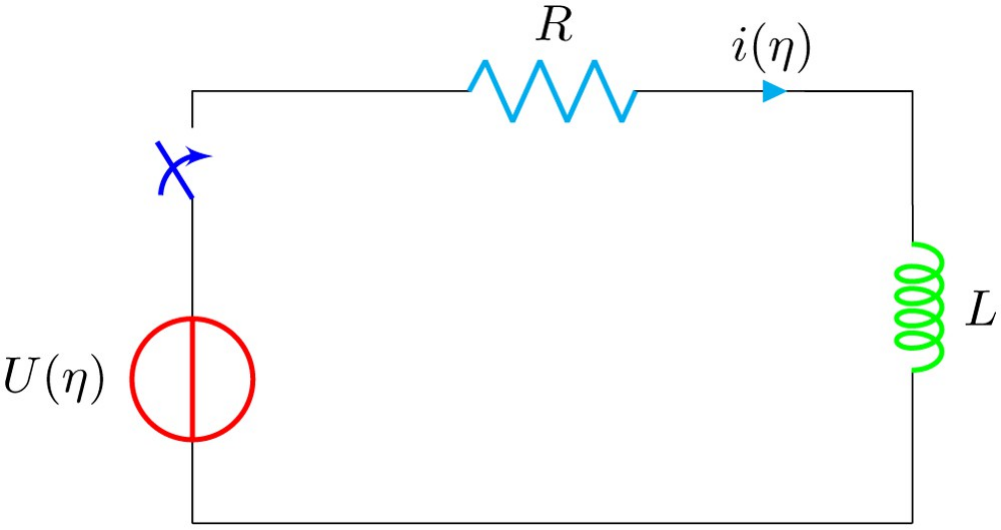
RL circuit with nonlinear inductor.

Fractional-order circuit components, such as capacitors and inductors, have grown in popularity because they can better simulate frequency-dependent behavior than standard integer-order models. The fractional-order RL circuit, in particular, has been applied in a variety of domains, including power electronics and control systems. However, the addition of a nonlinear inductor complicates the mathematical description of the circuit, necessitating the development of specific numerical approaches. One such approach that has been deployed is the Jacobi collocation method **JCM**. Fig 1 illustrates the fractional-order resistor-inductor (RL) electric circuit with a non-linear inductor, as stated in the references [12, 13]. The approach of nonlocal Riccati, resistor-inductor circuit problem was investigated by applying the integro spline quasi-interpolation technique in [14].

In this work, we aim to study one of the famous problems in electricity and electrical engineering: the Riccati equation. The design and analysis of the RL circuit shown in the Fig 1 are modeled by the nonlinear initial value Riccati problem with fractional derivative.

Firstly, let us define the general form of the Riccati equation with fractional derivative as follows:

$$\begin{array}{c} \sum_{k=1}^{m}\;\mu_{k}\left(\eta \right)\;\Xi^{\left(k\;\alpha \right)}\left(\eta \right)=\zeta \left(\eta \right)+\xi \left(\eta \right)\;\Xi \left(\eta \right)+\vartheta \left(\eta \right)\;\Xi^{2}\left(\eta \right),\;m-1<m\;\alpha \leq m,\;0\leq \eta \leq 1, \end{array}$$ (1.1)

with

$$\begin{array}{c} \Xi^{\left(\kappa \right)}\left(0\right)=\delta_{\kappa }\;\kappa =0,1,\ldots ,\left\lceil m\alpha \right\rceil -1. \end{array}$$ (1.2)

In the realm of RL circuits, fractional-order elements can be understood as generalized inductors or resistors exhibiting memory effects. A fractional-order inductor can be seen as a device that not only resists alterations in current but also retains a memory of previous current values. A fractional-order resistor is a component whose resistance is contingent upon the historical voltage applied across it.

Motivation for Fractional-Order Modeling

- Memory effects: Real-world components frequently demonstrate memory effects, wherein their current behavior is influenced by prior situations. Fractional-order models can proficiently encapsulate these memory effects, resulting in more precise descriptions of circuit dynamics.
- Non-perfect components: Conventional models frequently presume perfect components, which may not consistently hold true in practical situations. Fractional-order models can include non-ideal phenomena, such as frequency-dependent resistance or inductance, offering a more accurate representation of circuit behavior.
- Complex dynamics: Fractional-order differential equations can demonstrate a broader spectrum of dynamic behaviors compared to their integer-order equivalents. This enhanced flexibility facilitates more precise modeling of intricate systems, especially those exhibiting chaotic or oscillatory behavior.

Fractional Riccati equations are commonly encountered in diverse fields such as applied sciences, engineering, and real-world applications. These equations are relevant in areas such as diffusion problems, control theory, pattern generation in dynamic gas systems, network synthesis, river flows, invariant embedding, and econometric models [33]. Because of the Riccati differential equation’s relevance and numerous applications, scientists have done countless investigations to find more efficient and precise methods for solving it. Several analytical and numerical techniques have arisen in the literature to tackle this problem. A short survey considering these approaches that have been proposed in Table 2.

**Table 2:** A short survey of the recent researches related to the Riccati IVP.

No.	Numerical Method	Author	Year	Ref.
1	Enhanced Homotopy perturbation technique	Ranjbar et al.	2008	[34]
2	Homotopy analysis approach	Tan et al.	2008	[35]
3	Modified variation iteration method (MVIM)	Geng	2010	[36]
4	Homotopy perturbation technique	Khan et al.	2011	[37]
5	Fractional variational iteration	Merdan	2012	[38]
6	Reproducing kernel Hilbert space	Sakar et al.	2017	[39]
7	Bezier curves technique	Ghomanjani et al.	2017	[40]
8	Chebyshev polynomials	Eldien et al.	2019	[41]
9	Laplace transform by	Liu et al.	2020	[42]
10	A three-point clock multistep technique	Rasdee et al.	2020	[43]
11	Homotopy analysis Sumudu transform method (HASTM)	Singh et al.	2023	[44]

**Fig 2:**
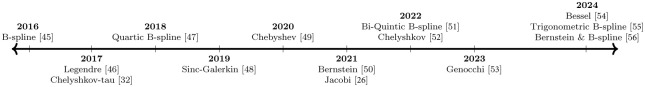
Timeline of the recent contributions in collocation techniques [26, 32, 45–56].

The collocation technique offers an extremely efficient spectral approach that may be flexibly utilized to estimate the approximate solution of ordinary and partial differential equations at predefined points in the solution domain. The unknown function is represented as a truncated series of basis functions, with coefficients determined by solving equations. The type of equations to be solved depends on the characteristics of the initial problem and the collocation methods utilized. El-Gamel and his research group have widely contributed to this field over the last ten years. They developed novel strategies and techniques based on various basis functions to approximate the solutions of differential, integral, and integro-differential equations in engineering applications. A brief timeline of the main papers is arranged in the diagram shown in Fig 2.

This paper contains multiple original contributions to the analysis and resolution of RL electric circuits represented by fractional Riccati initial value problems (IVPs). We provide a novel implementation of the fractional Riccati model to appropriately represent the intricate memory-dependent behavior of RL circuits, marking a substantial improvement over conventional integer-order models that frequently inadequately characterize these systems. Additionally, we offer a novel numerical technique that integrates the Jacobi collocation method with a Broyden-modified Newton algorithm. This hybrid Jacobi-Broyden Newton method facilitates the efficient and precise solution of the nonlinear fractional differential equation by utilizing the operational matrices of Jacobi polynomials. This integration improves computing efficiency and guarantees superior convergence rates, as rigorously evidenced by error and residual assessments. This study enhances existing numerical approaches and applies them to a more realistic fractional model, surpassing previous research in both theoretical and practical dimensions of resolving RL circuit issues, hence paving the way for further exploration in fractional-order electrical systems. This paper offers multiple original contributions to the analysis and resolution of RL electric circuits represented by fractional Riccati initial value problems (IVPs). We provide a novel use of the fractional Riccati model to effectively capture the intricate memory-dependent behavior of RL circuits, representing a substantial improvement over conventional integer-order models that frequently inadequately characterize these systems. Additionally, we offer a novel numerical technique that integrates the Jacobi collocation method with a Broyden-modified Newton algorithm. This hybrid Jacobi-Broyden Newton method facilitates the efficient and precise solution of the nonlinear fractional differential equation by utilizing the operational matrices of Jacobi polynomials. This integration improves computing efficiency and guarantees superior convergence rates, as rigorously evidenced by error and residual assessments. This study enhances existing numerical approaches and applies them to a more realistic fractional model, surpassing previous research in both theoretical and practical dimensions of solving RL circuit issues, hence paving the way for further exploration in fractional-order electrical systems.

The organization of this manuscript is structured as follows: Some preliminary material contains the basic definitions, lemmas, and theorems related to fractional derivatives, and the basic steps of Jacobi’s polynomials and their operational matrix are indeed accessible in the second section. Section 3 deals with the derivation of the discrete system obtained from the Jacobi collocation method. Section 4 derives the residual error of the proposed technique. Section 5 reveals numerical simulations and comparisons with other methods. Finally, the conclusions are given in Section 6.

## 2 Preliminaries and relations

In the following section, the basic theorems and relations that will be utilized in the paper will be considered.

### 2.1 Fractional derivative

**Definition 2.1** *The Caputo fractional integral* IC0,ηα *for the real function* Ξ(*η*) *is represented by* [6]

IC0,ηα[Ξ(η)]=1Γ(α)∫0η(η-τ)α-1Ξ(η)dτ,α>0η>0.

**Definition 2.2** *The fractional derivative operator of Caputo type* DC0,ηα *for function* Ξ(*η*) *with order α* > 0 *is defined by* [6]

DC0,ηα[Ξ(η)]=1Γ(n-α)∫0η(η-τ)n-α-1Ξ(n)(η)dτ,n-1<α≤n,n∈N,

*where*

-
$\Gamma \left(n\right)=\int_{0}^{\infty }\;\eta^{n-1}e^{-\eta }\;d\eta$ ; *Gamma function*,
- Ξ^(*n*)^(*η*) *is the classical n*^*th*^ *derivatives with respect to η*.

**Proposition 2.3** [57] *Assume that* DC0,ηα[Ξ(η)] *exists, then*

limα→nDC0α[Ξ(η)]=Ξ(n)(η),limα→n-1DC0α[Ξ(η)]=Ξ(n-1)(η)-Ξ(n-1)(0).

**Lemma 2.4** *If η*^*μ*^ *is a polynomial of μ*− *degree and* $N_{0}\equiv N\cup 0$, *then*

DC0,ηα[ημ]={0,forμ∈N0andμ<⌈α⌉,Γ(μ+1)Γ(μ+1-α)ημ-α,forμ∈N0orμ∉Nandμ≥⌈α⌉.

where *α*, *μ* ≥ 0 and ⌈*α*⌉ is ceiling function.

### 2.2 An overview of Jacobi polynomials

**Definition 2.5** *Jacobi polynomial* $\Psi_{K}^{\left(\theta_{1},\theta_{2}\right)}\left(\eta \right),\;\theta_{1},\;\theta_{2}>-1$ *and η* ∈ [−1, 1] *is given by*

Ψi(θ1,θ2)(η)=∑i=0n2-i(K+θ1+θ2+ii)(K+θ1K-i)(η-1)i.

Jacobi polynomials have the following properties [58].

- Orthogonal polynomials over the interval *η* ∈ [−1, 1] with respect to the weight *w*(*η*).
  $$\begin{array}{c} w\left(\eta \right)={\left(1-\eta \right)}^{\theta_{1}}{\left(1+\eta \right)}^{\theta_{2}}, \end{array}$$ (2.1)
  $$\begin{array}{c} \int_{-1}^{1}\Psi_{n}^{\left(\theta_{1},\theta_{2}\right)}\left(\eta \right)\Psi_{m}^{\left(\theta_{1},\theta_{2}\right)}\left(\eta \right){\left(1-\eta \right)}^{\theta_{1}}{\left(1+\eta \right)}^{\theta_{2}}d\eta =\\ \;\;\frac{2^{\theta_{1}+\theta_{2}+1}}{2n+\theta_{1}+\theta_{2}+1}\frac{\gamma \left(n+\theta_{1}+1\right)\gamma \left(n+\theta_{2}+1\right)}{\gamma (n+\theta_{1}+\theta_{2}+1)}\delta_{n,m}. \end{array}$$
- The Rodrigues formula
  $${\left(1-\eta \right)}^{\theta_{1}}{\left(1+\eta \right)}^{\theta_{2}}\Psi_{K}^{\left(\theta_{1},\theta_{2}\right)}\left(\eta \right)=\frac{{\left(-1\right)}^{K}}{2^{K}K!}\frac{d^{K}}{d\eta^{K}}[{\left(1-\eta \right)}^{K+\theta_{1}}{\left(1+\eta \right)}^{K+\theta_{2}}],$$
-
$\Psi_{0}^{\left(\theta_{1},\theta_{2}\right)}\left(\eta \right)=1$ , $\Psi_{1}^{\left(\theta_{1},\theta_{2}\right)}\left(\eta \right)=\frac{\theta_{1}+\theta_{2}+2}{2}\eta +\frac{\theta_{1}-\theta_{2}}{2}$.
-
$\Psi_{K}^{\left(\theta_{1},\theta_{2}\right)}\left(-\eta \right)={\left(-1\right)}^{K}\Psi_{K}^{\left(\theta_{1},\theta_{2}\right)}\left(\eta \right)$ .

*θ*_1_ and *θ*_2_ significantly influence the characteristics of the Jacobi polynomials. Here are some special cases of Jacobi polynomials based on the values of *θ*_1_ and *θ*_2_ [59].

- Legendre polynomials *P*_*K*_(*η*): when the Laplacian is split in spherical coordinates as functions of the polar angle *β* with (*η* = cos *β*).
- First kind Chebyshev polynomials *T*_*K*_(*η*):
  $$T_{K}\left(\eta \right)=\Psi_{K}^{\left(\frac{-1}{2},\frac{1}{2}\right)}\left(\eta \right)/\Psi_{K}^{\left(\frac{-1}{2},\frac{1}{2}\right)}\left(1\right).$$
- Second kind Chebyshev polynomials *U*_*n*_(*η*):
  $$U_{n}\left(\eta \right)=\left(K+1\right)\Psi_{K}^{\left(\frac{1}{2},\frac{1}{2}\right)}\left(\eta \right)/\Psi_{K}^{\left(\frac{1}{2},\frac{1}{2}\right)}\left(1\right).$$
- Gegenbauer polynomials *G*_*K*_(*η*) or ultraspherical polynomials are Jacobi polynomials with equal parameters.

For more flexibility, the Jacobi polynomials are redefined over the interval [0, 1]; they will be denoted by shifted Jacobi polynomials.

**Definition 2.6** *The shifted Jacobi polynomial is a polynomial of degree i defined over the range η* ∈ [0, 1] *and is defined by* [60]

$$\begin{array}{c} \Psi_{i}^{\left(\theta_{1},\theta_{2}\right)}\left(\eta \right)=\sum_{j=0}^{i}\;{\left(-1\right)}^{i-j}\frac{\Gamma \left(\theta_{2}+i+1\right)\Gamma \left(\theta_{1}+\theta_{2}+i+j+1\right)}{\Gamma \left(\theta_{2}+j+1\right)\Gamma \left(\theta_{1}+\theta_{2}+i+1\right)\left(i-j\right)!j!}\;\eta^{j}. \end{array}$$ (2.2)

### 2.3 Convergence of Jacobi polynomial

In this part, we examine the convergence and error bounds for Jacobi polynomials. The following three lemmas establish the upper limit of approximation error, as demonstrated in [61]. To derive an upper bound on approximation error, assume the function Ξ has the first *n* + 1 continuous derivatives, i.e. Ξ ∈ *C*^*n*+1^[0, 1].

**Lemma 2.7** *Suppose that the function* Ξ(η):[0,1]→R *has n* + 1 *continuous derivatives*, Ξ ∈ *C*^*n*+1^[0, 1] *and* Ξ_*n*_ *is the best approximation of* Ξ *in the space* X *where*

$$X=Span\;\{\Psi_{0}^{\left(\theta_{1},\theta_{2}\right)}\left(\eta \right),\Psi_{1}^{\left(\theta_{1},\theta_{2}\right)}\left(\eta \right),\Psi_{2}^{\left(\theta_{1},\theta_{2}\right)}\left(\eta \right),\ldots ,\Psi_{n}^{\left(\theta_{1},\theta_{2}\right)}\left(\eta \right)\},$$

*then*

$$\|\Xi -\Xi_{n}{\|}_{\Psi^{\left(\theta_{1},\theta_{2}\right)}}\leq \frac{M}{(n+1)!}\sqrt{\frac{\Gamma \left(1+\theta_{1}\right)\Gamma \left(2n+\theta_{2}+3\right)}{\Gamma (2n+\theta_{1}+\theta_{2}+4)}},$$

*where M* = max|Ξ^(*n*+1)^(*η*)| *over* 0 ≤ *η* ≤ 1.

Lemma 2.7 demonstrates that the optimal approximation approaches Ξ as the derivative Ξ satisfies the continuity requirement, as *n* tends towards infinity.

**Definition 2.8** *The function* Ξ *on* [0, 1] *has modulus of continuity* $\bar{\omega }\left(\Xi ,\delta \right)$ *which is defined as*

$$\bar{\omega }\left(\Xi ,\delta \right)=\text{sup}|\Xi \left(\eta \right)-\Xi \left(\overset{˘}{\eta }\right)|,$$

*where* $\eta ,\overset{˘}{\eta }\in \left[0,1\right],\;|\eta -\overset{˘}{\eta }|\leq \delta$.

**Lemma 2.9** Ξ(*η*) *is uniformly continuous on* [0, 1] *iff* $\underset{\delta \to 0}{\text{lim}}\;\bar{\omega }\left(\Xi ,\delta \right)=0$.

**Lemma 2.10** *Suppose that* Ξ(*η*) *is bounded on* [0, 1] *and* Ξ *in the space* X *then*

$$\|\Xi -\Xi_{n}{\|}_{2}\leq \;\frac{3}{2}\;\bar{\omega }\left(\Xi ,\frac{1}{\sqrt{n}}\right),$$

*we have* $\underset{n\to \infty }{\text{lim}}\;\bar{\omega }\left(\Xi ,\frac{1}{\sqrt{n}}\right)=0$, *then* $\underset{n\to \infty }{\text{lim}}$ Ξ_*n*_(*η*) = Ξ(*η*).

## 3 Jacobi-Collocation scheme

**Fig 3:**
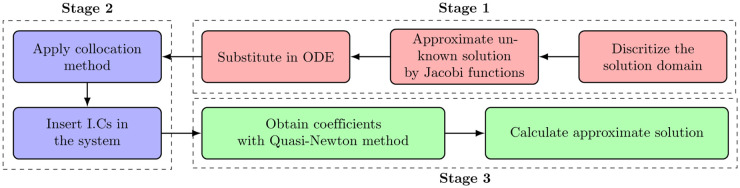
Jacobi Collocation Procedure (JCM).

Jacobi Collocation Method (**JCM**) is developed to obtain an approximate solution for the nonlinear fractional Riccati problem Eq (1.1), along with the given initial conditions (1.2). The full algorithm is divided into three stages and explained step by step in Fig 3.

To apply the collocation scheme for calculating in the solution of (1.1), the function Ξ(*η*) is approximated in terms of a truncated shifted Jacobi polynomial series as follows:

$$\begin{array}{c} \Xi \left(\eta \right)≅\Xi_{n}\left(\eta \right)=\sum_{i=0}^{n}\;\varrho_{i}\;\Psi_{i}^{\left(\theta_{1},\theta_{2}\right)}\left(\eta \right), \end{array}$$ (3.1)

where $\Psi_{i}^{\left(\theta_{1},\theta_{2}\right)}\left(\eta \right),\;\eta \in \left[0,1\right]$ are the orthogonal shifted Jacobi polynomials and $\varrho_{i}$ are unknown coefficients which can be calculated via the following form:

$$\varrho_{i}=\frac{1}{\psi_{i}}\int_{0}^{1}\;w\left(\eta \right)\;\Xi \left(\eta \right)\;\Psi_{i}^{\left(\theta_{1},\theta_{2}\right)}\left(\eta \right)d\eta \;i=0,1,\cdots n.$$

In the above formula, *w*(*η*) are the weights given by Eq (2.1) and *ψ*_*i*_ are represented in the following expanded form for simplicity:

$$\psi_{i}=\frac{\Gamma \left(i+\theta_{1}+1\right)\Gamma \left(i+\theta_{2}+1\right)}{\left(2i+\theta_{1}+\theta_{2}+1\right)i!\Gamma \left(i+\theta_{1}+\theta_{2}+1\right)}.$$

Eq (3.1) is written as follows

$$\begin{array}{c} \Xi (\eta )=\Psi (\eta )\;\Pi , \end{array}$$ (3.2)

where

$$\begin{array}{c} \Pi =[\varrho_{0},\ldots ,\varrho_{n}{]}^{⊤},\;\Psi \left(\eta \right)=[\begin{array}{ccccc} \Psi_{0}^{\left(\theta_{1},\theta_{2}\right)}\left(\eta \right) & \Psi_{1}^{\left(\theta_{1},\theta_{2}\right)}\left(\eta \right) & \Psi_{2}^{\left(\theta_{1},\theta_{2}\right)}\left(\eta \right) & \ldots & \Psi_{n}^{\left(\theta_{1},\theta_{2}\right)}\left(\eta \right) \end{array}]. \end{array}$$ (3.3)

**Lemma 3.1** [62] *If the shifted Jacobi polynomial* $\Psi_{i}^{\left(\theta_{1},\theta_{2}\right)}\left(\eta \right)$ *is defined in* (2.2) *then*

$$\begin{array}{c} \frac{d\Xi }{d\eta }=\sum_{i=0}^{n}\;\varrho_{i}[\frac{d}{d\;\eta }\;\Psi_{i}^{\left(\theta_{1},\theta_{2}\right)}\left(\eta \right)]. \end{array}$$ (3.4)

*And the derivatives in* (3.4) *can be written as*

$$\begin{array}{c} {}[\Xi^{\prime }\left(\eta \right)]=\Psi \left(\eta \right)\;D\;\Pi , \end{array}$$ (3.5)

*such that* ***D***^*T*^ *is a square matrix has* (*n* + 1) *rows and* (*n* + 1) *columns and can be written as follows*:

$$D^{⊤}=\left(d_{ij}\right)=\{\begin{array}{cc} \hat{d}\left(i,j\right) & if\;i>j,\\ 0 & otherwise, \end{array}$$

*where* [63]

d^(i,j)=(θ1+θ2+i+1)(θ1+θ2+i+2)j(θ1+j+2)i-j-1Γ(θ1+θ2+j+1)(i-j-1)!Γ(θ1+θ2+2j+1)×F32(-i+j+1,θ1+θ2+i+j+2,θ1+j+1;1θ1+j+2,θ1+θ2+2j+2),

**Lemma 3.2** [62] *If* $\Psi_{i}^{\left(\theta_{1},\theta_{2}\right)}\left(\eta \right)$ *defined in* (2.2) *is the shifted Jacobi polynomial then*

$$\begin{array}{c} D^{\left(\alpha \right)}\Psi_{i}^{\left(\theta_{1},\theta_{2}\right)}\left(\eta \right)=0,\;i=0,1,\ldots ,\left\lceil \alpha \right\rceil -1. \end{array}$$ (3.6)

**Lemma 3.3** [62] *Let* $\Psi_{i}^{\left(\theta_{1},\theta_{2}\right)}\left(\eta \right)$ *be shifted Jacobi polynomials defined in* (2.2) *then*

$$\begin{array}{c} \begin{array}{cc} \Xi^{\left(\alpha \right)}\left(\eta \right) & =\sum_{i=0}^{n}\;\varrho_{i}[\Psi_{i}^{\left(\theta_{1},\theta_{2}\right)}\left(\eta \right){]}^{\left(\alpha \right)},\;\;\;\;\\ \left[\Xi^{\left(\alpha \right)}\left(\eta \right)\right] & =\Psi \left(\eta \right)\;D^{\left(\alpha \right)}\;\Pi , \end{array} \end{array}$$ (3.7)

*where operational matrix of fractional derivative is* ***D***^(*α*)^ *with dimensions* (*n* + 1) × (*n* + 1)

$$\begin{array}{c} D^{\left(\alpha \right)}=[\begin{array}{ccccc} 0 & 0 & 0 & \ldots & 0\\ \vdots & \vdots & \vdots & \ldots & \vdots \\ 0 & 0 & 0 & \ldots & 0\\ \Delta_{\alpha }\left(\left\lceil \alpha \right\rceil ,0\right) & \Delta_{\alpha }\left(\left\lceil \alpha \right\rceil ,1\right) & \Delta_{\alpha }\left(\left\lceil \alpha \right\rceil ,2\right) & \ldots & \Delta_{\alpha }\left(\left\lceil \alpha \right\rceil ,n\right)\\ \vdots & \vdots & \vdots & \ldots & \vdots \\ \Delta_{\alpha }\left(i,0\right) & \Delta_{\alpha }\left(i,1\right) & \Delta_{\alpha }\left(i,2\right) & \ldots & \Delta_{\alpha }\left(i,n\right)\\ \vdots & \vdots & \vdots & \ldots & \vdots \\ \Delta_{\alpha }\left(n,0\right) & \Delta_{\alpha }\left(n,1\right) & \Delta_{\alpha }\left(n,2\right) & \ldots & \Delta_{\alpha }\left(n,n\right) \end{array}{]}^{⊤}, \end{array}$$ (3.8)

*and*

$$\Delta_{\alpha }\left(i,j\right)=\sum_{p=\lceil \alpha \rceil }^{i}\;\varsigma_{i,j,p},$$

*and*

$$\begin{array}{c} \begin{array}{c} \varsigma_{i,j,p}=\frac{{\left(-1\right)}^{i-p}\Gamma \left(\theta_{2}+j+1\right)\Gamma \left(\theta_{2}+i+1\right)\Gamma \left(\theta_{1}+\theta_{2}+i+p+1\right)}{\psi_{j}\Gamma \left(\theta_{1}+\theta_{2}+j+1\right)\Gamma \left(\theta_{2}+p+1\right)\Gamma \left(\theta_{1}+\theta_{2}+i+1\right)\Gamma \left(p-\alpha +1\right)\left(i-p\right)!}\\ \times \sum_{l=0}^{j}\;\frac{{\left(-1\right)}^{j-l}\Gamma \left(\theta_{1}+\theta_{2}+j+l+1\right)\Gamma \left(\theta_{1}+1\right)\Gamma \left(\theta_{2}+l+p-\alpha +1\right)}{\Gamma \left(\theta_{2}+l+1\right)\Gamma \left(\theta_{1}+\theta_{2}+l+p-\alpha +2\right)\left(j-l\right)!\left(l!\right)}. \end{array} \end{array}$$ (3.9)

**Lemma 3.4** [64]

$$\begin{array}{c} \begin{array}{cc} \left[\begin{array}{c} \Xi^{\upsilon }\left(\eta_{0}\right)\\ \Xi^{\upsilon }\left(\eta_{1}\right)\\ \vdots \\ \Xi^{\upsilon }\left(\eta_{n}\right) \end{array}\right] & ={\left[\begin{array}{cccc} \Xi \left(\eta_{0}\right) & 0 & \cdots & 0\\ 0 & \Xi \left(\eta_{1}\right) & \cdots & 0\\ \vdots & \vdots & \; & \vdots \\ 0 & 0 & \cdots & \Xi \left(\eta_{n}\right) \end{array}\right]}^{\upsilon -1}\left[\begin{array}{c} \Xi \left(\eta_{0}\right)\\ \Xi \left(\eta_{1}\right)\\ \vdots \\ \Xi \left(\eta_{n}\right) \end{array}\right],\\ & ={\tilde{\Xi }}^{\upsilon -1}\;\Xi ,\\ & ={\left(\tilde{\Psi }\;\;\tilde{\Pi }\right)}^{\upsilon -1}\;\Psi \;\Pi , \end{array} \end{array}$$ (3.10)

where

$$\tilde{\Pi }=[\begin{array}{cccc} \Pi & 0 & \cdots & 0\\ 0 & \Pi & \cdots & 0\\ \vdots & \vdots & \; & \vdots \\ 0 & 0 & \cdots & \Pi \end{array}],\;\tilde{\Psi }=[\begin{array}{cccc} \Psi (\eta_{0}) & 0 & \cdots & 0\\ 0 & \Psi (\eta_{1}) & \cdots & 0\\ \vdots & \vdots & \; & \vdots \\ 0 & 0 & \cdots & \Psi (\eta_{n}) \end{array}],$$

*and*

$$\Psi =[\begin{array}{c} \Psi (\eta_{0})\\ \Psi (\eta_{1})\\ \vdots \\ \Psi (\eta_{n}) \end{array}]=[\begin{array}{ccccc} \Psi_{0}^{\left(\theta_{1},\theta_{2}\right)}\left(\eta_{0}\right) & \Psi_{1}^{\left(\theta_{1},\theta_{2}\right)}\left(\eta_{0}\right) & \Psi_{2}^{\left(\theta_{1},\theta_{2}\right)}\left(\eta_{0}\right) & \ldots & \Psi_{n}^{\left(\theta_{1},\theta_{2}\right)}\left(\eta_{0}\right)\\ \Psi_{0}^{\left(\theta_{1},\theta_{2}\right)}\left(\eta_{1}\right) & \Psi_{1}^{\left(\theta_{1},\theta_{2}\right)}\left(\eta_{1}\right) & \Psi_{2}^{\left(\theta_{1},\theta_{2}\right)}\left(\eta_{1}\right) & \ldots & P_{n}^{\left(\alpha ,\beta \right)}\left(\eta_{1}\right)\\ \vdots & \vdots & \ldots & \vdots \\ \Psi_{0}^{\left(\theta_{1},\theta_{2}\right)}\left(\eta_{n}\right) & \Psi_{1}^{\left(\theta_{1},\theta_{2}\right)}\left(\eta_{n}\right) & \Psi_{2}^{\left(\theta_{1},\theta_{2}\right)}\left(\eta_{n}\right) & \ldots & \Psi_{n}^{\left(\theta_{1},\theta_{2}\right)}\left(\eta_{n}\right) \end{array}].$$

By substitute Eqs (3.1) in (1.1) we obtian

$$\begin{array}{c} \sum_{k=1}^{m}\;\mu_{k}\left(\eta \right)[\;\sum_{i=0}^{n}\;\varrho_{i}{\left[\Psi_{i}^{\left(\theta_{1},\theta_{2}\right)}\left(\eta \right)\right]}^{\left(k\;\alpha \right)}]=\zeta \left(\eta \right)+\xi \left(\eta \right)\;\sum_{i=0}^{n}\;\varrho_{i}\;\Psi_{i}^{\left(\theta_{1},\theta_{2}\right)}\left(\eta \right)\\ +\vartheta \left(\eta \right)\;[\sum_{i=0}^{n}\;\varrho_{i}\Psi_{i}^{\left(\theta_{1},\theta_{2}\right)}\left(\eta \right){]}^{2},\;m-1<m\;\alpha \leq m,\;0\leq \eta \leq 1. \end{array}$$ (3.11)

Now, we may substitute the collocation points *η*_*j*_, where *j* = 0, 1, …, *n*, with the roots of $\Psi_{n+1}^{\left(\theta_{1},\theta_{2}\right)}\left(\eta \right)$ in Eq (3.11). As a result, we obtain

$$\begin{array}{c} \sum_{k=1}^{m}\;\mu_{k}\left(\eta \right)[\;\sum_{i=0}^{n}\;\varrho_{i}{\left[\Psi_{i}^{\left(\theta_{1},\theta_{2}\right)}\left(\eta_{j}\right)\right]}^{\left(k\;\alpha \right)}]=\zeta \left(\eta_{j}\right)+\xi \left(\eta_{j}\right)\;\sum_{i=0}^{n}\;\varrho_{i}\;\Psi_{i}^{\left(\theta_{1},\theta_{2}\right)}\left(\eta_{j}\right)\\ +\vartheta \left(\eta_{j}\right)\;[\sum_{i=0}^{n}\;\varrho_{i}\Psi_{i}^{\left(\theta_{1},\theta_{2}\right)}\left(\eta_{j}\right){]}^{2},\;m-1<m\;\alpha \leq m. \end{array}$$ (3.12)

The next theorem will be obtained:

**Theorem 3.5** *If* (1.1) *is the estimated solution of the fractional Riccati differential equations* (3.1), *then the problem is reduced to solve the following nonlinear system for calculating the unknown coefficients*.

$$\begin{array}{c} Q\;\Pi =G, \end{array}$$ (3.13)

*where*

$$Q=\{\sum_{k=1}^{m}\mu_{k}\Psi \;[D^{\left(k\alpha \right)}]-\xi \;\Psi -\vartheta \;\bar{\Psi }\;\;\bar{\Pi }\;\Psi \}.$$

*and*

$$G=[\begin{array}{c} \zeta (\eta_{0})\\ \zeta (\eta_{1})\\ \zeta (\eta_{2})\\ \vdots \\ \zeta (\eta_{n}) \end{array}],\;\;\mu_{k}=[\begin{array}{ccccc} \mu_{k}\left(\eta_{0}\right) & 0 & 0 & \ldots & 0\\ 0 & \mu_{k}\left(\eta_{1}\right) & 0 & \ldots & 0\\ 0 & 0 & \mu_{k}\left(\eta_{2}\right) & \ldots & 0\\ \vdots & \vdots & \vdots & \ddots & \vdots \\ 0 & 0 & 0 & \ldots & \mu_{k}\left(\eta_{n}\right) \end{array}],$$

$$\xi =[\begin{array}{ccccc} \xi (\eta_{0}) & 0 & 0 & \ldots & 0\\ 0 & \xi (\eta_{1}) & 0 & \ldots & 0\\ 0 & 0 & \xi (\eta_{2}) & \ldots & 0\\ \vdots & \vdots & \vdots & \ddots & \vdots \\ 0 & 0 & 0 & \ldots & \xi (\eta_{n}) \end{array}],\;\;\;\vartheta =[\begin{array}{ccccc} \vartheta (\eta_{0}) & 0 & 0 & \ldots & 0\\ 0 & \vartheta (\eta_{1}) & 0 & \ldots & 0\\ 0 & 0 & \vartheta (\eta_{2}) & \ldots & 0\\ \vdots & \vdots & \vdots & \ddots & \vdots \\ 0 & 0 & 0 & \ldots & \vartheta (\eta_{n}) \end{array}].$$

**proof**: Each term in Eq (1.1) is replaced with its corresponding approximation from Eqs (3.2), (3.5), (3.7), and (3.10), while substituting *η* = *η*_*i*_. The initial conditions are obtained from Eq (1.2) and will be represented using matrices.

$$\begin{array}{c} \Psi \left(0\right)D^{\kappa }\Pi =\delta_{\kappa }\;\;\kappa =0,1,\ldots ,\left\lceil m\alpha \right\rceil -1. \end{array}$$ (3.14)

Moreover, the matrix form of the initial conditions becomes

$$\begin{array}{c} \Theta_{\kappa }\Pi =\delta_{\kappa }\;\;\text{or}\;\;\left[\Theta_{\kappa };\delta_{\kappa }\right];\;\;\kappa =0,1,\ldots ,\left\lceil m\alpha \right\rceil -1, \end{array}$$ (3.15)

where

$$\Theta_{\kappa }=[\begin{array}{cccc} \theta_{\kappa 0} & \theta_{\kappa 1} & \ldots & \theta_{\kappa n} \end{array}]=\Psi \left(0\right){\text{D}}^{\kappa }\Pi ,\;\;\kappa =0,1,\ldots ,\left\lceil m\alpha \right\rceil -1.$$

Consequently, in the augmented matrix [**Q**; **G**] if we replace the ⌈*mα*⌉ rows by the row matrices [Θ_*κ*_; *δ*_*κ*_],then the final system is obtained

$$\begin{array}{c} \left[\overset{˘}{\text{Q}}\;;\overset{˘}{\text{G}}\right]\;\;\text{or}\;\;\overset{˘}{\text{Q}}\;\Pi =\overset{˘}{\text{G}}. \end{array}$$ (3.16)

The unknown coefficients in the above nonlinear system of equations can be determined by utilizing the Quasi-Newton Broyden’s approach. The main steps of the scheme are summarized in Algorithm 1.

**Algorithm 1: JCM scheme of solution**

• Input *n*, *θ*_1_ and *θ*_2_.

• Define the *n* + 1 collocation points ${\left\{\eta_{i}\right\}}_{i=0}^{n}$ over the interval [*a*, *b*].

• Approximate the solution Ξ(*η*) via truncated Lucas series: $\Xi \left(\eta \right)=\sum_{s=0}^{n}\varrho_{s}\Psi_{s}^{\left(\theta_{1},\theta_{2}\right)}\left(\eta \right).$

• Substitute the approximate series representation of Ξ(*η*) into BVP.

• Apply the collocation points ${\left\{\eta_{i}\right\}}_{i=0}^{n}$ to the differential equation to obtain a discrete system.

• Incorporate the boundary equations into the discrete system.

• Use the quasi-Newton Broyden’s algorithm to solve the obtained nonlinear system for the unknowns ${\left\{\varrho_{s}\right\}}_{s=0}^{N}$.

### 3.1 Quasi-Newton Broyden’s algorithm

The quasi-Newton Broyden’s method uses the traditional iterative technique with some modifications to speed up the execution time to reach the equilibrium point. At the beginning, the system of nonlinear (*n* + 1) equations will be written in the following form:

$$\begin{array}{c} \omega \left(\Pi \right)=(\begin{array}{c} \omega_{0}\left(\varrho_{0},\varrho_{1},\ldots ,\varrho_{n}\right)\\ \omega_{1}\left(\varrho_{0},\varrho_{1},\ldots ,\varrho_{n}\right)\\ \vdots \\ \omega_{N}\left(\varrho_{0},\varrho_{1},\ldots ,\varrho_{n}\right) \end{array})=(\begin{array}{c} 0\\ 0\\ \vdots \\ 0 \end{array}), \end{array}$$ (3.17)

where

- **Π** is the unknowns’ vector.
- ***ω*** is the equations’ vector.

Let **Π**^(*k*)^ represent the estimated values of the unknown coefficients for the *k*th iteration. Let ***ω***^(*k*)^ denote the value of ***ω*** at the *k*^*th*^ iteration. Given that the magnitude of ‖***ω***^(*k*)^‖ is sufficiently enough, we seek to update the vectors Δ**Π**^(*k*)^.

$$\begin{array}{c} \Pi^{\left(k+1\right)}=\Pi^{\left(k\right)}+\Delta \Pi^{\left(k\right)}\Leftrightarrow (\begin{array}{c} \varrho_{0}^{\left(k+1\right)}\\ \varrho_{1}^{\left(k+1\right)}\\ \vdots \\ \varrho_{n}^{\left(k+1\right)} \end{array})=(\begin{array}{c} \varrho_{0}^{\left(k\right)}\\ \varrho_{1}^{\left(k\right)}\\ \vdots \\ \varrho_{n}^{\left(k\right)} \end{array})+(\begin{array}{c} \Delta \varrho_{0}^{\left(k\right)}\\ \Delta \varrho_{1}^{\left(k\right)}\\ \vdots \\ \Delta \varrho_{n}^{\left(k\right)} \end{array}), \end{array}$$ (3.18)

such that the vector *ω* evaluated at **Π**^(*k*+1)^ is equal to zero. Applying the multidimensional extension of Taylor expansion to approximate the change in ***ω***(**Υ**) near **Π** yields:

$$\begin{array}{c} \omega \left(\Pi^{\left(k\right)}+\Delta \;\Pi^{\left(k\right)}\right)=\omega \left(\Pi^{\left(k\right)}\right)+\omega^{\prime }\left(\Pi^{\left(k\right)}\right)\Delta \;\Pi^{\left(k\right)}+O\left({\left\|\Delta \;\Pi^{\left(k\right)}\right\|}^{2}\right), \end{array}$$ (3.19)

where ***ω***′(**Π**^(*k*)^) is the system’s Jacobian matrix.

$$\begin{array}{c} \omega^{\prime }\left(\Pi \right)\equiv \text{J}\left(\Pi \right)=(\begin{array}{cccc} \frac{\partial \omega_{0}}{\partial \varrho_{0}} & \frac{\partial \omega_{0}}{\partial \varrho_{1}} & \ldots & \frac{\partial \omega_{0}}{\partial \varrho_{n}}\\ \frac{\partial \omega_{1}}{\partial \varrho_{0}}) & \frac{\partial \omega_{1}}{\partial \varrho_{1}} & \ldots & \frac{\partial \omega_{1}}{\partial \varrho_{n}}\\ \vdots & \vdots & \vdots & \vdots \\ \frac{\partial \omega_{n}}{\partial \varrho_{0}} & \frac{\partial \omega_{n}}{\partial \varrho_{1}} & \ldots & \frac{\partial \omega_{n}}{\partial \varrho_{n}} \end{array}). \end{array}$$ (3.20)

If the higher order terms and neglected and by assuming that, **J**^(*k*)^ is the Jacobian at **Π**^(*k*)^ then Eq (3.19) will be reduced to:

$$\begin{array}{c} \omega \left(\Pi^{k}+\Delta \;\Pi^{k}\right)=\omega \left(\Pi^{\left(k\right)}\right)+{\text{J}}^{\left(k\right)}\Delta \;\Pi^{\left(k\right)}. \end{array}$$ (3.21)

By enforcing ***ω***(**Π**^(*k*)^ + Δ**Π**^(*k*)^) toward zero, then we have:

$$\begin{array}{c} {\text{J}}^{\left(k\right)}\Delta \;\Pi^{\left(k\right)}=-\omega \left(\Pi^{\left(k\right)}\right), \end{array}$$ (3.22)

It is unnecessary to compute the value of **J** at each iteration step due to the excessive time it consumes. Instead, the Jacobian will be revised by employing the subsequent formula:

$${\text{J}}^{\left(k\right)}={\text{J}}^{\left(k-1\right)}+[\frac{\Delta \;\omega \left(\Pi^{\left(k\right)}\right)-{\text{J}}^{\left(k-1\right)}\Delta \;\Pi^{\left(k\right)}}{\Delta^{⊤}\;\Pi^{\left(k\right)}\Delta \;\Pi^{\left(k\right)}}]\Delta^{⊤}\;\Pi^{\left(k\right)}.$$

If the absolute difference between two successive iterations is less than a user-defined tolerance, i.e., when ∥**Π**^(*k*+1)^ − **Π**^(*k*)^∥ ≤ *ε*, then the process will be terminated.

**Algorithm 2: Quasi-Newton Broyden’s**

• Give an initial **Π** = **Π**^(0)^.

• Calculate ***J***^(0)^, ***ω***^(0)^, **Π**^(1)^.

• For *k* = 1, 2, 3, …

• Find ***ω***^(*k*)^, Δ***ω***^(*k*)^, Δ**Π**^(**k**)^.

• If ‖***ω***^(*k*)^‖ is small enough, stop.

• Calculate ${\text{J}}^{\left(k\right)}={\text{J}}^{\left(k-1\right)}+[\frac{\Delta \;\omega^{\left(k\right)}-{\text{J}}^{\left(k-1\right)}\Delta \;\Pi^{\left(k\right)}}{\Delta^{⊤}\Pi^{\left(k\right)}\Delta \;\Pi^{\left(k\right)}}]\Delta^{⊤}\Pi^{\left(k\right)}$.

• Solve ***J***^(*k*)^ Δ**Π**^(*k*)^ = −***ω***^(*k*)^.

• Compute **Π**^(*k*+1)^ = **Π**^(*k*)^ + Δ**Π**^(*k*)^.

• End.

## 4 Residual error estimation

The residual error function was used to estimate errors in the Jacobi-collocation approach. [65]. Consider the residual function *ς*_*n*_(*η*) is:

$$\begin{array}{c} \varsigma_{n}\left(\eta \right)=\sum_{k=1}^{m}\;\mu_{k}\left(\eta \right)\;{\hat{\Xi }}^{\left(k\;\alpha \right)}\left(\eta \right)-\zeta \left(\eta \right)-\xi \left(\eta \right)\;\hat{\Xi }\left(\eta \right)-\vartheta \left(\eta \right)\;{\hat{\Xi }}^{2}\left(\eta \right), \end{array}$$ (4.1)

where $\hat{\Xi }\left(\eta \right)$ is the **JCM** approximate solution (3.1) for Eqs (1.1) and (1.2). The following equation will be obtained if we subtract the Eqs (4.1) from (1.1).

$$\begin{array}{c} \sum_{k=1}^{m}\;\mu_{k}\left(\eta \right)\;[\Xi^{\left(k\;\alpha \right)}\left(\eta \right)-{\hat{\Xi }}^{\left(k\;\alpha \right)}\left(\eta \right)]+\xi \left(\eta \right)\;[\Xi \left(\eta \right)-\hat{\Xi }\left(\eta \right)]+\vartheta \left(\eta \right)\;[\Xi^{2}\left(\eta \right)-{\hat{\Xi }}^{2}\left(\eta \right)]=-\varsigma_{n}\left(\eta \right). \end{array}$$ (4.2)

Then the linear and nonlinear terms are manipulated, respectively as follows:

$$\ell \left[\varepsilon \left(\xi \right)\right]=\ell [\Xi \left(\eta \right)-\hat{\Xi }\left(\eta \right)]=\sum_{k=1}^{m}\;\mu_{k}\left(\eta \right)\;\varepsilon^{\left(k\;\alpha \right)}\left(\eta \right)+\xi \left(\eta \right)\;\varepsilon \left(\eta \right),$$

such that *ε*(*η*) is the function of error and Ξ(*η*) is the exact solution of Eq (1.1),

$$\begin{array}{c} ℵ\;\left[\Xi \left(\eta \right)\right]=\vartheta \left(\eta \right)\;\Xi^{2}\left(\eta \right), \end{array}$$ (4.3)

$$\begin{array}{c} ℵ\;\left[\hat{\Xi }\left(\eta \right)+\varepsilon \left(\eta \right)\right]=\vartheta \left(\eta \right)[\hat{\Xi }\left(\eta \right)+\varepsilon \left(\eta \right){]}^{2}. \end{array}$$ (4.4)

Eq (4.2) can be written in the following operator form:

$$\begin{array}{c} \ell \;\left[\varepsilon \left(\eta \right)\right]+ℵ\;\left[\Xi \left(\eta \right)\right]-ℵ\;\left[\hat{\Xi }\left(\eta \right)\right]=-\varsigma_{n}\left(\eta \right). \end{array}$$ (4.5)

Consequently,

$$\begin{array}{c} \begin{array}{c} \sum_{k=1}^{m}\;\mu_{k}\left(\eta \right)\;\varepsilon^{\left(k\;\alpha \right)}\left(\eta \right)+\xi \left(\eta \right)\;\varepsilon \left(\eta \right)+\vartheta \left(\eta \right)[\varepsilon^{2}\left(\eta \right)+2\varepsilon \left(\eta \right)\hat{\Xi }\left(\eta \right)]=-\varsigma_{n}\left(\eta \right),\\ \varepsilon^{\left(\kappa \right)}\left(0\right)=0\;\kappa =0,1,\ldots ,\left\lceil m\alpha \right\rceil -1, \end{array} \end{array}$$ (4.6)

the above equation has the following solution:

$$\varepsilon \left(\eta \right)=\sum_{i=0}^{n}{\tilde{\varrho }}_{i}\Psi_{i}^{\left(\theta_{1},\theta_{2}\right)}\left(\eta \right).$$

By applying the proposed algorithm in Section 3, the unknown values ${\tilde{\varrho }}_{i},\;i=0,1,2,\ldots n$ are obtained. The maximum absolute errors will be calculated by:

$$\begin{array}{c} E_{\text{max}}=\underset{0<\eta <1}{\text{max}}\left|\varepsilon (\xi )\right|. \end{array}$$ (4.7)

Through the utilization of maximum error estimate, we may assess the dependability of the outcomes, particularly when an exact solution is not available.

## 5 Results and simulations

In this section, we first provide three test problems to test the **JCM** for solving the Riccati problem with various nonlinear terms. Furthermore, we compare our methods, namely **JCM** at *θ*_1_ = *θ*_2_ = 0 in all examples with other methods introduced in previous works [18, 33, 66, 67]. In the second part, the application of the RL problem is illustrated and solved with **JCM** to test the proposed algorithm and showcase its accuracy and reliability.

### 5.1 Test problems

**Example 1** [66] Assume problem (1.1) with its parameters as follows *ζ*(*η*) = 1, *ξ*(*η*) = 0, *ϑ*(*η*) = −1, *μ*_1_(*η*) = 1 and *μ*_*k*_(*η*) = 0 for *k* = 2, 3, ⋯, *m*.

$$\frac{d^{\alpha }\Xi }{d\eta^{\alpha }}+\Xi^{2}\left(\eta \right)=1,\;\Xi \left(0\right)=0\;0\leq \eta \leq 1.$$

For the sake of calculating error bounds, we use the exact solution at *α* = 1 in the form:

$$\Xi \left(\eta \right)=\frac{e^{2\eta }-1}{e^{2\eta }+1}.$$

In Tables 3–5, a comprehensive comparison is presented for the numerical solution obtained using the following techniques; Joint Collocation Method (JCM) in conjunction with the improved Adams-Bashforth-Moulton method (IABMM) [66], the modified homotopy perturbation method (MHPM) [18], the enhanced homotopy perturbation method (EHPM) [66] and the Bernstein collocation method (BCM) [67]. Table 3 denotes the values of the solutions for *α* = 0.75; Table 4 shows them for *α* = 0.9; and Table 5 gives the values of the solutions for *α* = 1.

**Table 3:** Comparison of the numerical solutions with the other methods for *α* = 0.75 and different choices of *n* for Example 1.

*η*	JMC			IABMM [66]	EHPM [66]	MHPM [18]	BCM [67]
	*n* = 5	*n* = 8	*n* = 11				
0.2	0.3427	0.3078	0.3022	0.3117	0.3214	0.3138	0.3099
0.4	0.5094	0.4778	0.4767	0.4855	0.5077	0.4929	0.4816
0.6	0.6122	0.5691	0.5944	0.6045	0.6259	0.5974	0.5977
0.8	0.6892	0.6668	0.6764	0.6880	0.7028	0.6604	0.6788
1.0	0.7439	0.7280	0.7350	0.7478	0.7542	0.7183	0.7368

**Table 4:** Numerical comparisons of techniques for *α* = 0.9 and varied *n* values in Example 1.

*η*	JMC			IABMM [66]	EHPM [66]	MHPM [18]	BCM [67]
	*n* = 5	*n* = 8	*n* = 11				
0.2	0.2479	0.2382	0.2353	0.2393	0.2647	0.2391	0.2387
0.4	0.4332	0.4221	0.4198	0.4234	0.4591	0.4229	0.4225
0.6	0.5734	0.5596	0.5640	0.5679	0.6031	0.5653	0.5661
0.8	0.6798	0.6705	0.6730	0.6774	0.7068	0.6740	0.6746
1.0	0.7584	0.7515	0.7534	0.7584	0.7806	0.7569	0.7545

**Table 5:** Comparison of numerical solutions with several approaches for *α* = 1 and various *n* selections in Example 1.

*η*	Exact solution	JCM (*n* = 5)	JCM (*n* = 11)	MHPM [18]	BCM [67]
0.2	0.1973753202	0.1975882276	0.1973753077	0.197375	0.197375
0.4	0.3799489622	0.3802576960	0.379948951	0.379944	0.379948
0.6	0.5370495669	0.5372758888	0.5370495581	0.536857	0.537049
0.8	0.6640367702	0.6642281778	0.6640367633	0.661706	0.664036
1.0	0.7615941559	0.7617340667	0.7615941506	0.746032	0.761594

**Fig 4:**
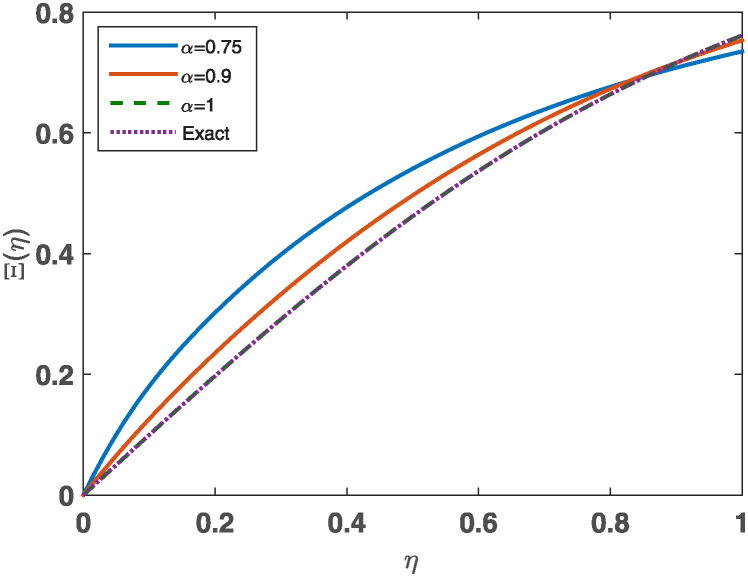
JCM’s approximate solutions with *n* = 11, *α* = 0.75, 0.9, 1. for Ex 1.

**Fig 5:**
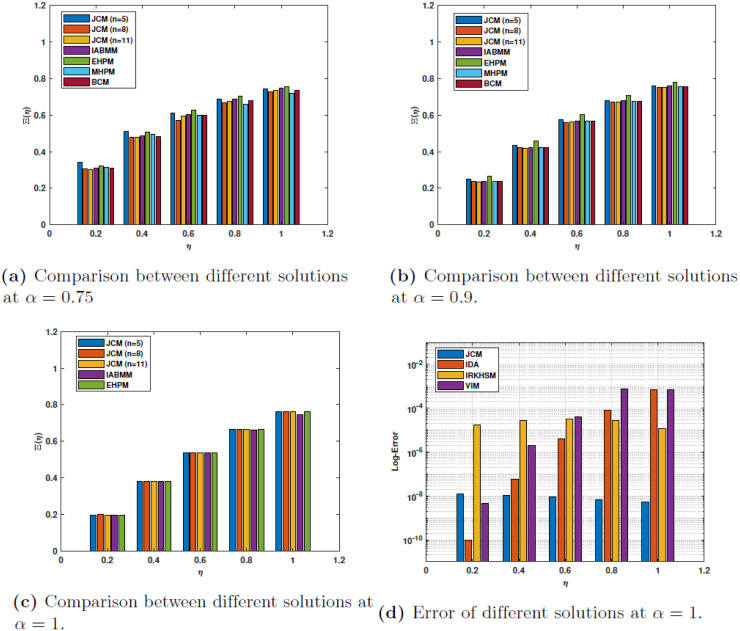
Comparison of the results for Example 1.

The absolute error for the case *α* = 1 obtained by our proposed method **JCM** is compared to the absolute errors derived from the variational iteration method **(VIM)**, the iterative decomposition algorithm **(IDA)**, and the iterative reproducing kernel Hilbert spaces method **(IRKHSM)** in Table 6. The numerical values demonstrate the superior precision of **JCM** compared to the other methods. Fig 4 illustrates the estimated solutions for *n* = 11, *α* = 0.75, 0.9, 1., specifically for example 1. However, the comprehensive outcomes are graphically represented as bar charts and may be found in Fig 5.

**Table 6:** Comparison of absolute errors for *α* = 1 in Example 1 using this approach and others.

*η*	JCM (*n* = 11)	IDA [68]	IRKHSM [69]	VIM [70]
0.2	1.2426E-08	1.00E-10	1.72E-05	4.39E-09
0.4	1.0822E-08	5.61E-08	2.85E-05	1.97E-06
0.6	8.8108E-09	4.09E-06	3.17E-05	4.09E-05
0.8	6.9363E-09	7.78E-05	2.81E-05	7.35E-04
1.0	5.2721E-09	6.99E-04	1.19E-05	6.99E-04

**Example 2**: [66] considered that *ζ*(*η*) = 1, *ξ*(*η*) = 2, *ϑ*(*η*) = −1, *μ*_1_(*η*) = 1 and *μ*_*k*_(*η*) = 0 for *k* = 2, 3, …, *m* in problem (1.1), then

$$\frac{d^{\gamma }\Xi }{d\eta^{\alpha }}=2\;\Xi \left(\eta \right)-\Xi^{2}\left(\eta \right)+1,\;\Xi \left(0\right)=0,\;0\leq \eta \leq 1.$$

The exact solution of the this problem for *α* = 1 is

$$\Xi \left(\eta \right)=1+\sqrt{2}\;\text{tanh}\;(\sqrt{2}\eta +\frac{1}{2}\text{ln}(\frac{\sqrt{2}-1}{\sqrt{2}+1})).$$

**Fig 6:**
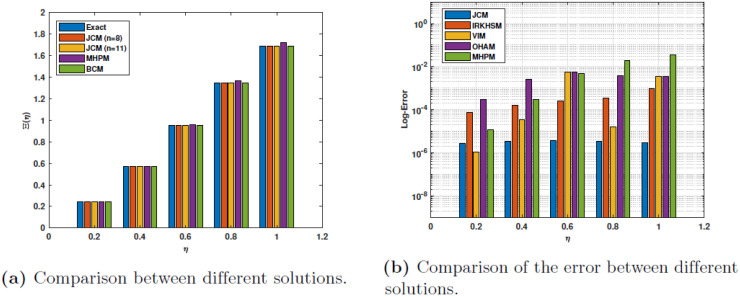
Comparison of the results at *α* = 1 for Example 2.

**Fig 7:**
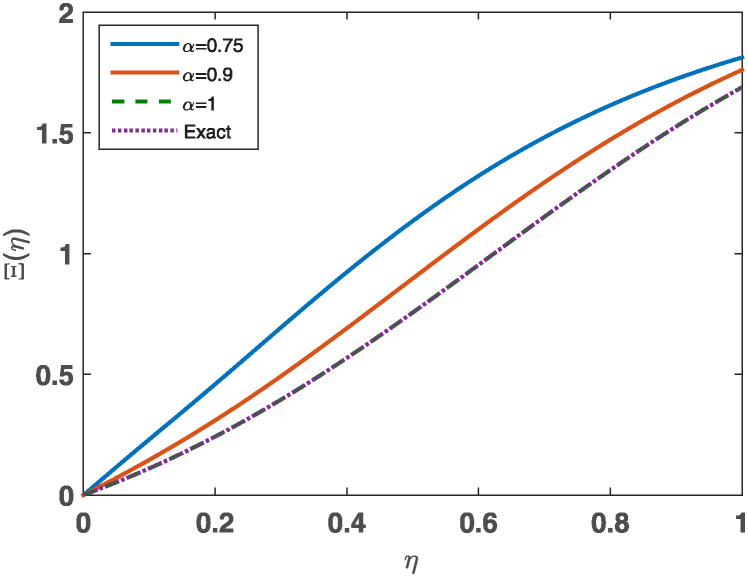
A plot of the approximate solutions for *n* = 11, *α* = 0.75, 0.9, 1. for Example 2.

The numerical results found in Table 7 are being compared with different approaches. [18, 66, 67] for *α* = 1. TThe comparison examines well-known methodologies: variational iteration method (VIM), modified homotopy perturbation method (MHPM), Optimal homotopy asymptotic method (OHAM), and iterative reproducing kernel Hilbert spaces method (IRKHSM). The current method’s absolute errors for *α* = 1 are compared with the absolute errors of other procedures in Table 8. The comparison of the approximate solution at certain places and the error from the JCM and other approaches are represented by Fig 6. The results are also presented in Tables 7 and 8. Fig 7 shows the **JCM**’s estimated solution for *n* = 11 at different values of the fractional derivative’s order: *α* = 0.75, 0.9, 1. for Example 2. Based on the data acquired, it is evident that the results produced by our suggested approach are more precise in comparison to the other outcomes.

**Table 7:** Comparison of the numerical solution via JCM with the other methods at *α* = 1 for Example 2.

*η*	Exact solution	JCM (*n* = 8)	JMC (*n* = 11)	MHPM [18]	BCM [67]
0.2	0.241976799	0.241976080	0.241976809	0.241965	0.241977035
0.4	0.567812166	0.567811952	0.567812176	0.568115	0.567812472
0.6	0.953566217	0.953533772	0.953566203	0.958259	0.953566555
0.8	1.346363655	1.346335424	1.346363610	1.365240	1.346363997
1.0	1.689498392	1.689473775	1.689500176	1.723810	1.689510190

**Table 8:** Examining the absolute errors of the current approach and alternative methods for *α* = 1 in Example 2.

*η*	JCM (*n* = 11)	IRKHSM [69]	VIM [70]	OHAM [71]	MHPM [18]
0.2	2.67E-06	7.58E-05	1.03E-06	2.90E-04	1.20E-05
0.4	3.35E-06	1.66E-04	3.33E-05	2.50E-03	3.03E-04
0.6	3.68E-06	2.52E-04	5.50E-03	5.50E-03	4.69E-03
0.8	3.46E-06	3.40E-04	1.54E-05	3.80E-03	1.88E-02
1.0	2.81E-06	9.22E-04	3.47E-03	3.40E-03	3.43E-02

**Example 3**: [33] Consider that *ζ*(*η*) = 0, *ξ*(*η*) = −1, *ϑ*(*η*) = 1, *μ*_1_(*η*) = 1 and *μ*_*k*_(*η*) = 0 for *k* = 2, 3, …, *m*, in problem (1.1), then we obtain:

$$\frac{d^{\alpha }\Xi }{d\eta^{\alpha }}+\Xi \left(\eta \right)-\Xi^{2}\left(\eta \right)=0,\;\Xi \left(0\right)=0.5,\;0\leq \eta \leq 1.$$

The exact solution for *α* = 1 of this problem is

$$\Xi \left(\eta \right)=\frac{e^{-\eta }}{e^{-\eta }+1}.$$

**Fig 8:**
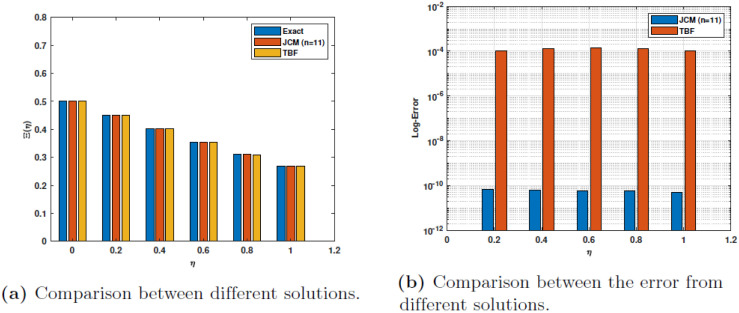
Comparison of the results at *α* = 1 for Example 3.

**Fig 9:**
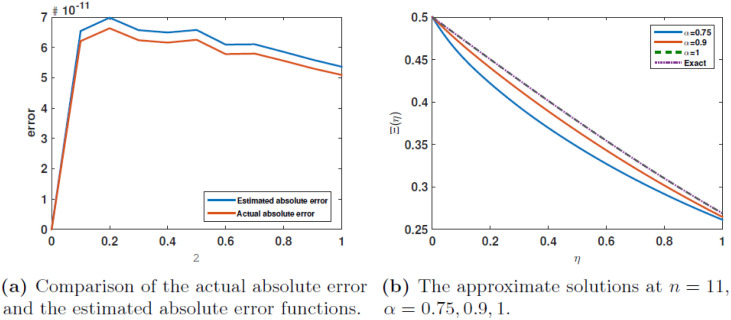
The results of Example 3.

The Table 9 presents a comparison between the numerical solutions obtained using our approach and the real absolute errors for *γ* = 1. The solutions and errors are also compared with those obtained using trigonometric basic functions (**TBF**). The current absolute errors for *α* = 1 are compared with the estimated absolute errors in Table 10. All these data appear in Tables 9 and 10 are displayed in Fig 8 for the comparisons of approximate solution and the absolute error. The results exhibit a clear pattern of consistency, with a significant disparity between the inaccuracy of JCM and TBF. Fig 9a illustrates the estimated solutions for *n* = 11, *α* = 0.75, 0.9, 1., specifically for the case of 3. The Fig 9b illustrates the contrast between the actual absolute error and the estimated absolute error functions.

**Table 9:** Numerical results of Example 3 with *α* = 1.

*η*	Exact solution	JCM (*n* = 11)	TBF [33]	Error in JCM (*n* = 11)	Error in TBF [33]
0.0	0.5	0.5	0.5	0	0
0.2	0.450166002	0.450166002	0.450065	6.63E-11	1.01E-04
0.4	0.401312339	0.401312339	0.401178	6.15E-11	1.34E-04
0.6	0.354343693	0.354343693	0.354203	5.77E-11	1.40E-04
0.8	0.310025518	0.310025518	0.309897	5.55E-11	1.28E-04
1.0	0.268941421	0.268941421	0.268837	5.09E-11	1.04E-04

**Table 10:** Illustration of the actual and estimated absolute errors of the current approach at *α* = 1 and *n* = 11 for Example 3.

*η*	(Estimated error)	(Actual error)
0.2	6.98E-11	6.63E-11
0.4	6.49E-11	6.15E-11
0.6	6.09E-11	5.77E-11
0.8	5.85E-11	5.55E-11
1.0	5.36E-11	5.09E-11

### 5.2 Analysis of LR circuit

Consider the RL circuit shown in Fig 1. The circuit with second-order non-linearity can be modeled by an initial value problem (IVP) [12, 72], as follows:

$$\begin{array}{c} \Xi^{\left(\alpha \right)}\left(\eta \right)+\sigma \;R\;\Xi^{2}\left(\eta \right)=U\left(\eta \right),\;\;0<\alpha \leq 1,\;0\leq \eta \leq 1, \end{array}$$ (5.1)

subject to initial conditions

$$\begin{array}{c} \Xi (0)=0. \end{array}$$ (5.2)

In Eq (5.1), The terms Ξ and *σ* respectively denote flux linkage in the inductor and the induction parameter. The non-linearity of the nonlinear inductor can be mathematically stated as:

$$\begin{array}{c} i\left(\Xi \right)=\sigma \;R\;\Xi^{2}, \end{array}$$ (5.3)

where *i*(Ξ) represents the current. For the transient analysis of the circuit shown in Fig 9, Kirchhoff’s second law is applied, which results in the following equation:

$$\begin{array}{c} \Xi^{\left(\alpha \right)}\left(\eta \right)+R\;i\left(\Xi \right)=U\left(\eta \right). \end{array}$$ (5.4)

JCM is used in a variety of circumstances where variable resistance, current, voltage, and inductance values are considered in nonlinear electrical circuit models. Assume the integer derivative order is *α* = 1.

In scenario **1**, a constant voltage magnitude of *U*(*η*) = 200*V* and inductance *σ* = 1 are utilized, while the resistance *R* is adjusted. The values of resistance, denoted by *R*, are 100 ohms, 125 ohms, and 155 ohms for instances 1, 2, and 3, respectively. The resistance *R* is varied while keeping voltage and inductance constant, we observe that higher resistance values lead to a decrease in flux linkage. This is consistent with physical intuition since increasing resistance limits current flow, subsequently reducing the magnetic flux produced in the inductor.

In scenario **2**, the analysis focuses on the changes in voltage *U*(*η*), while maintaining a constant resistance value of 100Ω and an inductance parameter of *σ* = 1. Three scenarios are examined using *U*(*η*) = 150*V*, 200*V*, and 250*V* for instances 1, 2, and 3, respectively. This case shows that if *U*(*η*) is variable with constant resistance and inductance, the results show that increasing voltage leads to a proportionate increase in flux linkage. This behavior is expected because higher input voltage generally drives higher current through the circuit, resulting in greater flux linkage.

In scenario **3** of the nonlinear RL circuits issue, the voltage *U*(*η*) is set to 200*V* and the resistance *R* is set to 100Ω, while the change in the inductance parameter *σ* is taken into account. Three examples are analyzed using standard deviations of 0.5, 1, and 1.5 for cases 1, 2, and 3, respectively. This scenario involves varying inductance while holding voltage and resistance constant. The results indicate that larger inductance values yield higher flux linkage. This makes sense as the inductor’s ability to store magnetic energy is directly proportional to its inductance.

In scenario **4**, a constant voltage magnitude of *U*(*η*) = 20 V and an inductance parameter of *σ* = 0.5 are utilized, while the resistance *R* is adjusted. The values of resistors, namely *R* = 1KΩ, 2KΩ, and 3KΩ, are being taken into account. This scenario examine a case with a high resistance *R* and a lower constant voltage, with variable resistance values. The results illustrate that even with smaller changes in resistance, flux linkage decreases as resistance increases. This demonstrates the circuit’s sensitivity to resistance when high resistance values are present, which may be particularly relevant for certain practical applications involving power dissipation.

**Fig 10:**
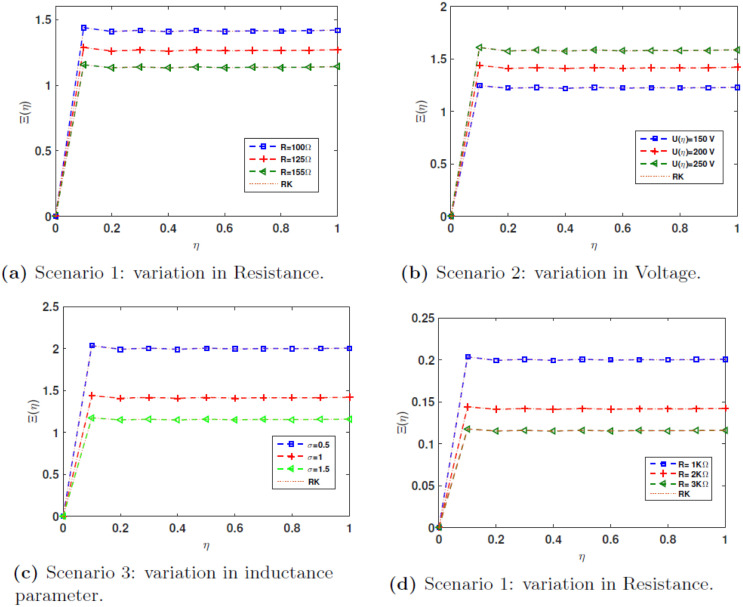
Comparison of results for the four scenarios based on nonlinear inductor circuit model.

Fig 10 depicts the approximate flux linkage of the inductor using **JCM**, which coincides with the results using the Runge-Kutta method for scenarios **1**, **2**, **3** and **4**.

**Fig 11:**
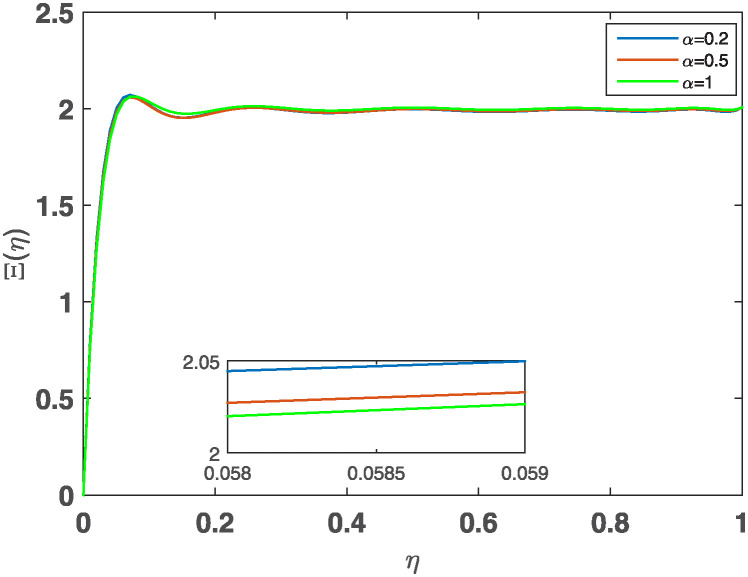
The behavior of the approximate the flux linkage of the inductor at various values *α*.

Fig 11 illustrates the behavior of the approximate flux linkage of the inductors at various values of the fractional parameter *α* = 0.2, 0.5, 1 for the RL circuit, while the values of *R* = 100Ω, the inductance parameter *σ* = 0.5 and the voltage *U*(*η*) = 200*V* are kept fixed. This figure illustrates the behavior of the approximate flux linkage of the inductor for different values of *α*. The observations are as follows:

- *α* = 0.2: The flux linkage rises sharply initially but then stabilizes at a lower value. This behavior suggests a system with a faster initial response and slower decay.
- *α* = 0.5: The curve shows a moderate rise and a slower decay compared to *α* = 0.2. This indicates a system with a balance between the initial response and decay.
- *α* = 1: The curve represents the standard integer-order RL circuit. It exhibits a relatively slower initial rise and faster decay compared to the fractional-order cases.

## 6 Conclusions and future work

This study introduces the shifted Jacobi-collocation approach as a means to get approximate solutions for fractional Riccati-type differential equations, which belong to the category of fractional differential equations. This technique converts the fractional Riccati-type differential problem into a set of nonlinear algebraic equations. The approximate answers can be derived by solving the resultant system. An appreciable benefit of this approach is that the answers may be readily acquired using computer tools like as MATLAB, MAPLE, and MATHEMATICA. The technique’s validity and usefulness are demonstrated through the inclusion of numerical examples. The aforementioned examples were executed on a computer with code written in MATLAB. The exhibited cases provide favorable outcomes, showcasing the efficacy of this strategy in achieving a high level of concordance between the approximate and precise values. The technique was utilized to investigate the relationship between flux and the variations in induction, resistance, and voltage values in the RL electric circuit. In summary, the Jacobi-collocation method is a highly appealing technique for solving fractional Riccati-type differential equations. It is capable of producing precise approximation solutions with the use of easily accessible computing resources. The RL circuit model derived from the Riccati equation can be generalized for various RL circuits, especially when incorporating fractional-order calculus. This includes the integration of nonlinear elements, fractional-order dynamics, and diverse boundary and initial conditions, such as circuits with time-varying voltage sources or initial magnetic fluxes, which can be accurately modeled by customizing these conditions. In conclusion, by altering parameters, boundary conditions, or incorporating supplementary terms, the Riccati equation framework employed herein can be effectively modified to model various RL and RLC circuits, serving as a robust instrument for the analysis of diverse nonlinear and fractional-order electrical systems. The RL circuit model derived from the Riccati equation can be generalized for various RL circuits, especially when incorporating fractional-order calculus. This includes the integration of nonlinear elements, fractional-order dynamics, and diverse boundary and initial conditions; for instance, circuits with time-varying voltage sources or initial magnetic fluxes can be accurately modeled by customizing these conditions. In conclusion, by altering parameters, boundary conditions, or incorporating supplementary terms, the Riccati equation framework employed here can be effectively modified to model various RL and RLC circuits, serving as a robust instrument for the analysis of diverse nonlinear and fractional-order electrical systems.

## References

[1] Bagley RL, Torvik PJ. Fractional calculus-a different approach to the analysis of viscoelastically damped structures. AIAA journal. 1983;21(5):741–748. doi: 10.2514/3.8142

[2] Gaul L, Klein P, Kempfle S. Impulse response function of an oscillator with fractional derivative in damping description. Mechanics Research Communications. 1989;16(5):297–305. doi: 10.1016/0093-6413(89)90067-0

[3] Caputo M. Linear models of dissipation whose Q is almost frequency independent. Annals of Geophysics. 1966;19(4):383–393.

[4] Debnath L. Recent applications of fractional calculus to science and engineering. International Journal of Mathematics and Mathematical Sciences. 2003;. doi: 10.1155/S0161171203301486

[5] Podlubny I. Fractional differential equations: an introduction to fractional derivatives, fractional differential equations, to methods of their solution and some of their applications. elsevier; 1998.

[6] Hilfer R. Applications of fractional calculus in physics. World scientific; 2000.

[7] Long LD, Moradi B, Nikan O, Avazzadeh Z, Lopes AM. Numerical approximation of the fractional Rayleigh–Stokes problem arising in a generalised Maxwell fluid. Fractal and Fractional. 2022;6(7):377. doi: 10.3390/fractalfract6070377

[8] Nikan O, Avazzadeh Z, Tenreiro Machado JA. Localized kernel-based meshless method for pricing financial options underlying fractal transmission system. Mathematical Methods in the Applied Sciences. 2024;47(5):3247–3260. doi: 10.1002/mma.7968

[9] Guo T, Nikan O, Avazzadeh Z, Qiu W. Efficient alternating direction implicit numerical approaches for multi-dimensional distributed-order fractional integro differential problems. Computational and Applied Mathematics. 2022;41(6):236. doi: 10.1007/s40314-022-01934-y

[10] Nikan O, Golbabai A, Machado JT, Nikazad T. Numerical approximation of the time fractional cable model arising in neuronal dynamics. Engineering with Computers. 2022;38(1):155–173. doi: 10.1007/s00366-020-01033-8

[11] Atangana A, Baleanu D. New fractional derivatives with nonlocal and non-singular kernel: theory and application to heat transfer model. arXiv preprint arXiv:160203408. 2016;.

[12] Köksal M, Herdem S. Analysis of nonlinear circuits by using differential Taylor transform. Computers & Electrical Engineering. 2002;28(6):513–525. doi: 10.1016/S0045-7906(00)00066-5

[13] Vazquez-Leal H, Boubaker K, Hernández-Martínez L, Huerta-Chua J. Approximation for transient of nonlinear circuits using RHPM and BPES methods. Journal of Electrical and Computer Engineering. 2013;2013(1):973813.

[14] Tanha N, Parsa Moghaddam B, Ilie M. Enhancing mathematical simulation: a novel integro spline quasi-interpolation for nonlocal dynamical systems. International Journal of Modelling and Simulation. 2024; p. 1–15. doi: 10.1080/02286203.2024.2327262

[15] Wang Q. Numerical solutions for fractional KdV–Burgers equation by Adomian decomposition method. Applied mathematics and computation. 2006;182(2):1048–1055. doi: 10.1016/j.amc.2006.05.004

[16] Inc M. The approximate and exact solutions of the space-and time-fractional Burgers equations with initial conditions by variational iteration method. Journal of Mathematical Analysis and Applications. 2008;345(1):476–484. doi: 10.1016/j.jmaa.2008.04.007

[17] Zhang X, Tang B, He Y. Homotopy analysis method for higher-order fractional integro-differential equations. Computers & Mathematics with Applications. 2011;62(8):3194–3203. doi: 10.1016/j.camwa.2011.08.032

[18] Odibat Z, Momani S. Modified homotopy perturbation method: application to quadratic Riccati differential equation of fractional order. Chaos, Solitons & Fractals. 2008;36(1):167–174. doi: 10.1016/j.chaos.2006.06.041

[19] Alchikh R, Khuri S. Numerical simulation of the fractional Lienard’s equation. International Journal of Numerical Methods for Heat & Fluid Flow. 2020;30(3):1223–1232. doi: 10.1108/HFF-06-2019-0458

[20] Matinfar M, Bahar SR, Ghasemi M. Solving the Lienard equation by differential transform method. World Journal of Modelling and Simulation. 2012;8(2):142–146.

[21] Meerschaert MM, Tadjeran C. Finite difference approximations for two-sided space-fractional partial differential equations. Applied numerical mathematics. 2006;56(1):80–90. doi: 10.1016/j.apnum.2005.02.008

[22] Karatay I, Bayramoğlu ŞR, Şahin A. Implicit difference approximation for the time fractional heat equation with the nonlocal condition. Applied Numerical Mathematics. 2011;61(12):1281–1288. doi: 10.1016/j.apnum.2011.08.007

[23] Li M, Gu XM, Huang C, Fei M, Zhang G. A fast linearized conservative finite element method for the strongly coupled nonlinear fractional Schrödinger equations. Journal of Computational Physics. 2018;358:256–282. doi: 10.1016/j.jcp.2017.12.044

[24] Zhang T, Guo Q. The finite difference/finite volume method for solving the fractional diffusion equation. Journal of Computational Physics. 2018;375:120–134. doi: 10.1016/j.jcp.2018.08.033

[25] Sazmand A, Behroozifar M. Application Jacobi spectral method for solving the time-fractional differential equation. Journal of Computational and Applied Mathematics. 2018;339:49–68. doi: 10.1016/j.cam.2018.02.018

[26] El-Gamel M, Abd El-Hady M. A fast collocation algorithm for solving the time fractional heat equation. SeMA Journal. 2021; p. 1–13.

[27] Abdelkawy MA, Lopes AM, Babatin MM. Shifted fractional Jacobi collocation method for solving fractional functional differential equations of variable order. Chaos, Solitons & Fractals. 2020;134:109721. doi: 10.1016/j.chaos.2020.109721

[28] Lakestani M, Dehghan M, Irandoust-Pakchin S. The construction of operational matrix of fractional derivatives using B-spline functions. Communications in Nonlinear Science and Numerical Simulation. 2012;17(3):1149–1162. doi: 10.1016/j.cnsns.2011.07.018

[29] Lepik Ü. Solving fractional integral equations by the Haar wavelet method. Applied Mathematics and Computation. 2009;214(2):468–478. doi: 10.1016/j.amc.2009.04.015

[30] Bhrawy AH, Alofi AS. The operational matrix of fractional integration for shifted Chebyshev polynomials. Applied Mathematics Letters. 2013;26(1):25–31. doi: 10.1016/j.aml.2012.01.027

[31] Chen J. A fast multiscale Galerkin algorithm for solving boundary value problem of the fractional Bagley–Torvik equation. Boundary Value Problems. 2020;2020:1–13. doi: 10.1186/s13661-020-01391-8

[32] El-Gamel M, Abd-El-Hady M, El-Azab Ms. Chelyshkov-tau approach for solving Bagley-Torvik equation. Applied Mathematics. 2017;8(12):1795. doi: 10.4236/am.2017.812128

[33] Agheli B. Approximate solution for solving fractional Riccati differential equations via trigonometric basic functions. Transactions of A Razmadze Mathematical Institute. 2018;172(3):299–308. doi: 10.1016/j.trmi.2018.08.002

[34] Ranjbar NAA, Hosseininia SH, Soltani I, Ghasemi J. A solution of Riccati nonlinear differential equation using enhanced homotopy perturbation method (EHPM). 2008;.

[35] Tan Y, Abbasbandy S. Homotopy analysis method for quadratic Riccati differential equation. Communications in Nonlinear Science and Numerical Simulation. 2008;13(3):539–546. doi: 10.1016/j.cnsns.2006.06.006

[36] Geng F. A modified variational iteration method for solving Riccati differential equations. Computers & Mathematics with Applications. 2010;60(7):1868–1872. doi: 10.1016/j.camwa.2010.07.017

[37] Khan NA, Ara A, Jamil M. An efficient approach for solving the Riccati equation with fractional orders. Computers & Mathematics with Applications. 2011;61(9):2683–2689. doi: 10.1016/j.camwa.2011.03.017

[38] Merdan M. On the Solutions Fractional Riccati Differential Equation with Modified Riemann-Liouville Derivative. International Journal of differential equations. 2012;2012(1):346089.

[39] Sakar MG, Akgül A, Baleanu D. On solutions of fractional Riccati differential equations. Advances in Difference Equations. 2017;2017:1–10. doi: 10.1186/s13662-017-1091-8

[40] Ghomanjani F, Khorram E. Approximate solution for quadratic Riccati differential equation. Journal of Taibah university for science. 2017;11(2):246–250. doi: 10.1016/j.jtusci.2015.04.001

[41] Ezz-Eldien SS, Machado JAT, Wang Y, Aldraiweesh AA. An algorithm for the approximate solution of the fractional Riccati differential equation. International Journal of Nonlinear Sciences and Numerical Simulation. 2019;20(6):661–674. doi: 10.1515/ijnsns-2018-0146

[42] Xin Liu K, Yao Y. Numerical Approximation of Riccati Fractional Differential Equation in the Sense of Caputo-Type Fractional Derivative. Journal of Mathematics. 2020;2020(1):1274251.

[43] bin Rasedee AFN, Sathar MHA, Ishak N, Hamzah SR, Jamaludin NA. Numerical Approximation of Riccati type differential equations. ASM Science Journal. 2020;.

[44] Singh J, Gupta A, Kumar D. Computational Analysis of the Fractional Riccati Differential Equation with Prabhakar-type Memory. Mathematics. 2023;11(3):644. doi: 10.3390/math11030644

[45] El-Gamel M, El-Shamy N. B-spline and singular higher-order boundary value problems. SeMA Journal. 2016;73:287–307. doi: 10.1007/s40324-016-0069-x

[46] El-Gamel M, Abd El-Hady M. Numerical solution of the Bagley-Torvik equation by Legendre-collocation method. SeMA Journal. 2017;74:371–383. doi: 10.1007/s40324-016-0089-6

[47] El-Gamel M, El-Shenawy A. A numerical solution of Blasius equation on a semi-infinity flat plate. SeMA Journal. 2018;75:475–484. doi: 10.1007/s40324-017-0145-x

[48] El-Gamel M, Abdrabou A. Sinc-Galerkin solution to eighth-order boundary value problems. SeMA Journal. 2019;76:249–270. doi: 10.1007/s40324-018-0172-2

[49] El-Gamel M, Mohamed O, El-Shamy N. A robust and effective method for solving two-point BVP in modelling viscoelastic flows. Applied Mathematics. 2020;11(01):23. doi: 10.4236/am.2020.111003

[50] El-Gamel M, Abd El-Hady M. On Using Bernstein Scheme for Computation of the Eigenvalues of Fourth-Order Sturm–Liouville Problems. International Journal of Applied and Computational Mathematics. 2021;7:1–18. doi: 10.1007/s40819-021-01059-6

[51] El-shenawy A, El-gamel M, Jaheen DR. Numerical Solution of Biharmonic Equation Using Modified Bi-Quintic B-Spline Collocation Method. MEJ Mansoura Engineering Journal. 2022;47(6):14–22. doi: 10.58491/2735-4202.3173

[52] El-Gamel M, El-Baghdady GI, Abd El-Hady M. Highly efficient method for solving parabolic PDE with nonlocal boundary conditions. Applied Mathematics. 2022;13(2):101–119. doi: 10.4236/am.2022.132009

[53] El-Gamel M, Mohamed N, Adel W. Genocchi collocation method for accurate solution of nonlinear fractional differential equations with error analysis. Mathematical Modelling and Numerical Simulation with Applications. 2023;3(4):351–375. doi: 10.53391/mmnsa.1373647

[54] El-shenawy A, gamel M E, Anany ME. A Novel Scheme Based on Bessel Operational Matrices for Solving a Class of Nonlinear Systems of Differential Equations. MEJ Mansoura Engineering Journal. 2024;49:1–10. doi: 10.58491/2735-4202.3192

[55] El-shenawy A, El-Gamel M, Reda D. Troesch’s problem: A numerical study with cubic trigonometric B-spline method. Partial Differential Equations in Applied Mathematics. 2024; p. 100694. doi: 10.1016/j.padiff.2024.100694

[56] El-Shenawy A, El-Gamel M, Abd El-Hady M. On the solution of MHD Jeffery–Hamel problem involving flow between two nonparallel plates with a blood flow application. Heat Transfer. 2024; p. 1–29.

[57] Atangana A. Fractional operators with constant and variable order with application to geo-hydrology. Academic Press; 2017.

[58] El-Gamel M, Kashwaa Y, Abd El-Hady M. Two highly accurate and efficient numerical methods for solving the fractional Liénard’s equation arising in oscillating circuits. Partial Differential Equations in Applied Mathematics. 2024;12:100914. doi: 10.1016/j.padiff.2024.100914

[59] Van Assche W. Ordinary Special Functions. In: Encyclopedia of Mathematical Physics. Oxford: Academic Press; 2006. p. 637–645.

[60] Singh H. Approximate solution of fractional vibration equation using Jacobi polynomials. Applied Mathematics and Computation. 2018;317:85–100. doi: 10.1016/j.amc.2017.08.057

[61] Behroozifar M, Sazmand A. An approximate solution based on Jacobi polynomials for time-fractional convection–diffusion equation. Applied Mathematics and Computation. 2017;296:1–17. doi: 10.1016/j.amc.2016.09.028

[62] Doha EH, Bhrawy AH, Ezz-Eldien SS. A new Jacobi operational matrix: an application for solving fractional differential equations. Applied Mathematical Modelling. 2012;36(10):4931–4943. doi: 10.1016/j.apm.2011.12.031

[63] Abd El-Hady M, El-shenawy A. Jacobi polynomials and the numerical solution of ray tracing through the crystalline lens. Optical and Quantum Electronics. 2024;56(8):1329. doi: 10.1007/s11082-024-07198-6

[64] Akyüz-Daşcıoğlu A, Çerdi H. The solution of high-order nonlinear ordinary differential equations by Chebyshev series. Applied Mathematics and Computation. 2011;217(12):5658–5666. doi: 10.1016/j.amc.2010.12.044

[65] Shahmorad S. Numerical solution of the general form linear Fredholm–Volterra integro-differential equations by the Tau method with an error estimation. Applied Mathematics and Computation. 2005;167(2):1418–1429. doi: 10.1016/j.amc.2004.08.045

[66] Hosseinnia SH, Ranjbar A, Momani S. Using an enhanced homotopy perturbation method in fractional differential equations via deforming the linear part. Computers & Mathematics with Applications. 2008;56(12):3138–3149. doi: 10.1016/j.camwa.2008.07.002

[67] Yüzbaşı Ş. Numerical solutions of fractional Riccati type differential equations by means of the Bernstein polynomials. Applied Mathematics and Computation. 2013;219(11):6328–6343. doi: 10.1016/j.amc.2012.12.006

[68] Taiwo OA, Osilagun JA. Approximate solution of generalized Riccati differential equations by iterative decomposition algorithm. Int J Eng Innovative Technol. 2012;1(2):53–56.

[69] Sakar MG. Iterative reproducing kernel Hilbert spaces method for Riccati differential equations. Journal of Computational and Applied Mathematics. 2017;309:163–174. doi: 10.1016/j.cam.2016.06.029

[70] Batiha B, Noorani M, Hashim I. Application of variational iteration method to a general Riccati equation. In: International mathematical forum. vol. 2; 2007. p. 2759–2770.

[71] Mabood F, Ismail AIM, Hashim I. Application of optimal homotopy asymptotic method for the approximate solution of Riccati equation. Sains Malaysiana. 2013;4:863–867.

[72] Mehmood A, Zameer A, Aslam MS, Raja MAZ. Design of nature-inspired heuristic paradigm for systems in nonlinear electrical circuits. Neural Computing and Applications. 2020;32:7121–7137. doi: 10.1007/s00521-019-04197-7

